# Trust Analysis Canvas for Teaching in the Field of Digital Public Health and Medicine: Tutorial

**DOI:** 10.2196/79709

**Published:** 2026-02-17

**Authors:** Federica Zavattaro, Clara-Maria Barth, Caroline Brall, Viktor von Wyl, Felix Gille

**Affiliations:** 1 Digital Society Initiative, University of Zurich, Rämistrasse 69, Zurich, 8001, Switzerland, +41 44 635 71 33; 2 Institute for Implementation Science in Health care (IfIS), University of Zurich, Zurich, Switzerland; 3 Department of Informatics, University of Zurich, Zurich, Switzerland; 4 Ethics and Policy Lab, Multidisciplinary Center for Infectious Diseases & Institute of Philosophy, University of Bern, Bern, Switzerland; 5 Epidemiology, Biostatistics & Prevention Institute (EBPI), University of Zurich, Zurich, Switzerland; 6 Swiss School of Public Health, Zurich, Switzerland

**Keywords:** trust, teaching, canvas, education, medical, digital health, medicine

## Abstract

Trust is increasingly recognized as a cornerstone for the successful implementation of digital public health initiatives, from mobile apps to the use of artificial intelligence in medicine, yet it remains underrepresented in educational curricula. In the course of our research and teaching activities in the field of trust in digital public health and medicine, we identified a gap in existing educational resources that aimed at supporting students in conducting structured trust analyses. Digitalization introduces new complexities into trust relationships, as interactions become increasingly mediated by digital tools. Preparing future professionals, therefore, demands fostering a critical understanding of how trust operates within digital systems, especially in the health sector. To address this gap, we developed and tested the first Trust Analysis Canvas for Teaching (TACT), a tool designed to guide students in conducting trust analyses of case studies in digital public health and medicine. Grounded in conceptual research on trust in health systems and health data sharing, we (1) developed the canvas content and reviewed it with two trust researchers; (2) tested and iteratively refined the tool with 23 students (3 BSc, 14 MSc, and 6 PhD) from diverse disciplines and academic levels through in-person and online focus groups at the universities of Zurich and Bern; (3) collaborated with a graphic designer to optimize its visual layout; and (4) translated the final canvas into French, Italian, German, and Spanish to ensure accessibility across disciplines, academic levels, and languages while maintaining a clear and engaging visual design. This paper introduces TACT, a canvas comprising 16 guiding questions organized around 6 core dimensions, designed to enable students from diverse disciplinary backgrounds and academic levels to engage with the complex concept of trust in a structured and guided manner, thereby addressing the identified gap in the current curricula. We outline the development process and provide a practical, step-by-step tutorial demonstrating its application through a written trust analysis of a digital health case study, supported by references to relevant literature.

## Introduction

Defined as “a bet about the future contingent actions of others” [1], trust has gained increasing momentum in digital health research and health policy making [2-4], where it is recognized as a central component of the relationship between the public and the health care system, enabling public participation and legitimizing health care activities [5]. Trust also plays a critical role in digital public health, understood as the integration of digital technologies into public health to enhance population health outcomes and efficiency of services [6], as well as in medicine and beyond. It shapes the design and use of medical applications [7], the adoption of electronic health records [8], vaccination uptake [9], compliance with nonpharmaceutical public health interventions [10], the use of e-government services [11], e-banking platforms [12], and artificial intelligence (AI) systems [13-15]. Across and beyond the health care sector, higher levels of trust are associated with greater social cohesion, lower system costs, improved service efficiency, and overall societal prosperity. Conversely, low levels of trust can undermine system stability, affecting sectors ranging from health care to government [5].

Trust operates within a context of vulnerability, in which the trustor relinquishes some control, lowers certain defenses, and exposes themselves to potential harm [16,17]. This is particularly relevant in digital public health and medicine, where digitalization enables the processing and sharing of large volumes of data, increasing both the complexity of trust relationships and the exposure of sensitive information to risks. In the context of health data—classified as sensitive personal data under the General Data Protection Regulation [18]—trust entails the expectation that institutions, organizations, and individuals responsible for collecting, storing, sharing, and processing health data will act with integrity, reliability, competence, and in the public’s best interest [5].

Preparing future professionals for these environments, therefore, requires more than technical expertise: students should also develop a critical understanding of how trust operates within digital systems. Despite its central importance, the current status of trust education in relevant university programs remains limited. In medicine, public health, and digital health curricula across bachelor’s (BSc), master’s (MSc), and doctoral levels (PhD), at least in our setting in Switzerland [19], there is no structured or systematic approach to teaching trust as an analytical concept, nor guidance on applying trust frameworks to real-world cases. While existing courses may touch on related dimensions such as ethics, they rarely provide dedicated space on the concept of trust itself. The limited availability of teaching materials in the literature that translate trust theory into practical, pedagogical formats suggests that this gap extends beyond our local context. In response, and in recognition of the growing societal significance of trust, we integrated applied case studies on trust into university teaching within the Digital Society Initiative PhD Excellence Program at the University of Zurich (UZH) [20]. A robust trust analysis requires a clear conceptual understanding of trust, as well as insight into the mechanisms that foster or undermine it in specific contexts. Relying solely on trust theories can be challenging for students. In disciplines such as ethics, the canvas format has proven effective in simplifying complex concepts. Defined as a “visual tool designed to guide people through the process of using a methodology or framework” [21], the canvas format has shown value in teaching and in fostering problem-solving and critical thinking [22,23]. To date, we have used ethics-focused tools that share some conceptual overlap with trust [21,24-26]. While these tools have proven useful for teaching and for guiding students through an analysis process, they were designed for ethical analysis specifically and therefore serve a different purpose.

In response to the lack of teaching aids on trust, this paper introduces the first Trust Analysis Canvas for Teaching (TACT), a tool designed to guide students in conducting trust analyses of case studies in digital public health and medicine. The objective was to develop a canvas that equips students from diverse disciplinary backgrounds and academic levels with skills relevant to their future careers by analyzing key societal challenges—ranging from the erosion of institutional trust to the strengthening of interpersonal relationships, including the doctor-patient relationship, and by enabling them to grasp high-level concepts of trust, navigate the complexities of trust analysis, and apply these insights effectively to real-world case studies in digital public health and medicine.

## Methods

### Development of TACT

The development of TACT followed a design science research approach, in which knowledge is generated through the iterative building and evaluation of purposeful artifacts [27]. We followed the 6 design science research activities between May 2024 and October 2024, progressing from the initial conceptualization of the canvas to the translation of its final version into multiple languages. Each activity is described in the corresponding sections of this paper.

### Activity 1: Problem Identification and Motivation

As outlined in the introduction, we identified a clear gap in existing teaching resources on trust and articulated the motivation for developing a structured tool to support trust analysis in educational settings.

### Activity 2: Definition of Objectives

As stated in the aims of the study, the objectives of TACT were to develop a tool that would enable students to conduct structured trust analyses of case studies in digital public health and medicine.

### Activity 3: Design and Development

FZ and FG developed the first version of TACT by integrating the “public trust in health data sharing” framework [28] and the “public trust in the health care system” framework [29] with theories that conceptualize trust as a relational, context-dependent, and inherently complex concept [1,5,30,31]. The conceptualization process was iterative: key trust dimensions from the selected frameworks were systematically reviewed, assessed against established trust theories, and translated into guiding questions for practical application in teaching contexts. Although the dimensions were initially derived from health-related frameworks, care was taken throughout the process to ensure they remained discipline-independent, allowing for their application across a broad range of case studies beyond the digital and public health domains. Once a preliminary set of 7 dimensions for conducting a trust analysis had been identified, FZ and FG tested the draft canvas internally using a fictitious case study, which led to an initial refinement. The canvas was then reviewed in writing by senior trust researchers, purposively selected for their expertise, and recruited via email by FG, to assess the conceptual robustness of the content. Their feedback informed further revisions, resulting in a refined second version (Multimedia Appendix 1) that was used for subsequent student testing and development.

### Activities 4 and 5: Demonstration and Evaluation

Following the pilot testing within the research group, FZ and FG conducted 3 English-language focus groups involving BSc, MSc, and PhD students from different disciplinary backgrounds. Focus groups were chosen to enable in-depth exploration of participants’ perceptions, capture interactive discussions on how TACT was interpreted and applied, and generate richer insights than individual interviews for its evaluation [32]. Reporting of the qualitative study followed the COREQ (Consolidated Criteria for Reporting Qualitative Research) guidelines (Checklist 1). PhD students from the UZH were purposively sampled based on their research areas; MSc students from the AI in Medicine program at the University of Bern participated as part of a course on Ethical and Legal issues in AI; and BSc students were recruited via UZH mailing lists. The canvas was tested both in person (with MSc students) using its printed version and online (with BSc and PhD students) using its digital version. Following each focus group, it was refined to incorporate participant feedback. The MSc cohort engaged with the canvas through a guided learning approach [33], while the BSc and PhD cohorts used a problem-based learning approach [34]; both methods were used in conjunction with digital public health and medicine case studies. The case study method was chosen for its capacity to promote critical thinking, conceptual integration, and collaboration [35]. The guided learning approach provides a structured learning experience through a strong conceptual foundation delivered by the lecturer [33]. The MSc focus group was held in person at the University of Bern (duration: 1 h 45 min). Students first received an introduction to the research objectives and an in-depth explanation of the concept of trust, then worked in small groups (2‐3 participants) to apply the canvas to a digital health case study (Multimedia Appendix 2), and participated in a debriefing plenary discussion. Two online focus groups (45 min each) were conducted via Microsoft Teams (version 4.19.82.0) with BSc and PhD students, using a problem-based learning approach, which is recognized for fostering deep conceptual understanding [34]. Students were given a brief digital public health case study and asked to engage with the canvas to conduct a trust analysis without prior theoretical instruction on trust. The case studies were tailored in complexity to suit each academic level (MultimediaAppendices 3  4). After 15 minutes of individual analysis, plenary discussions were held during which lecturers introduced the theoretical foundations of trust, facilitated the interpretation of the canvas, and gathered feedback and suggestions for improvement. FZ took notes during the focus group on the feedback received. The demonstration and evaluation phase concluded after focus groups with students from 3 academic levels were completed within the available timeframe. The consistency of feedback across groups indicated that further data collection was unlikely to yield additional value, giving us confidence that the key points requiring refinement had been adequately captured [36]. CMB, with expertise in graphic design, refined the canvas layout after each focus group based on student feedback, enhancing its visual intuitiveness and ensuring an engaging, user-friendly design. To overcome linguistic barriers and enhance both usability and applicability across countries, the canvas was translated from English into French, Italian, German, and Spanish. Each translation was carried out by 2 native speakers per language, all fluent in English.

### Activity 6: Communication and Dissemination

Communication and dissemination of the problem, the artifact, and evidence of its use to researchers and practitioners are achieved through the publication of this tutorial.

### Ethical Considerations

According to the Data Protection/Ethics Self-Assessment Tool of the University of Zurich’s Digital Society Initiative (2025-266615), the study does not fall under the Human Research Act and does not require submission to the Cantonal Ethics Committee. All participants provided written informed consent prior to participating in the focus groups, and no personal data were collected or processed during the study. Students received a CHF 20 (US $ 25.99) voucher as a token of appreciation. They were neither taught, supervised, evaluated, nor graded by FZ and FG and were not enrolled in any of their courses, ensuring the absence of any dependency or hierarchical relationship between researchers and participants.

## Results

### Study Participants

We gathered feedback from 2 trust researchers and 23 students at BSc, MSc, and PhD levels from diverse disciplinary backgrounds, through focus groups conducted both online and in-person, using both guided and problem-based learning approaches (Table 1). Students applied TACT to case studies in digital public health and medicine (Multimedia Appendices 2-4).

**Table 1.** Focus group participants (N=23 students).

Focus group	Participants, n (%)	Academic level	Discipline	Method
1	3 (13)	BSc students (University of Zurich)	Art, computational linguistics, and business administration	Online (guided learning approach)
2	14 (60.9)	MSc students (University of Bern)	Artificial intelligence in medicine	In-person (problem-based learning approach)
3	6 (26.1)	PhD students (University of Zurich)	Communication science, education, epidemiology, computer science, mathematics, and law	Online (guided learning approach)

### TACT

Feedback from trust researchers and students primarily concerned unclear phrasing and the layout of the tool. A comparison between the initial and final versions of the canvas highlights 5 key revisions (Multimedia Appendix 5). During testing, students reported that TACT effectively supported their understanding of trust under both guided and problem-based learning approaches, as it provided a logical structure for analyzing the various dimensions of trust within the case study.

**Figure 1.**
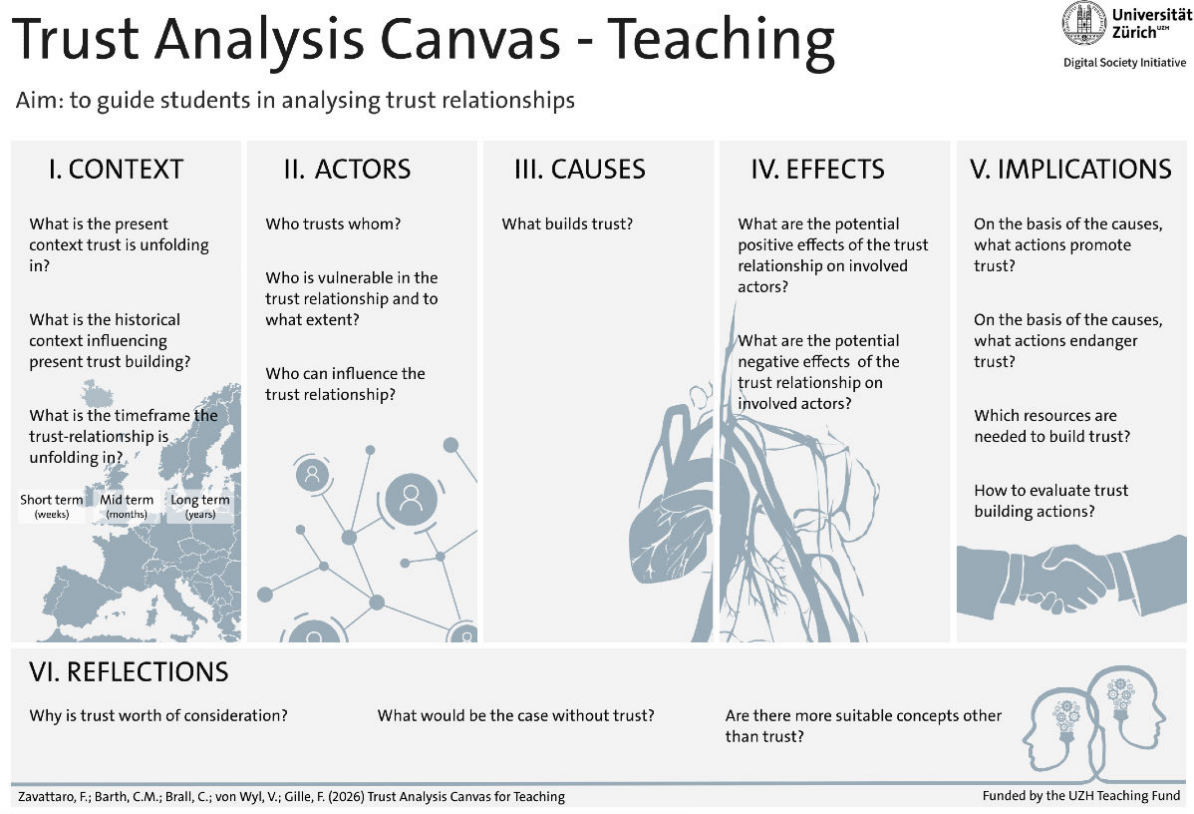
Trust Analysis Canvas for Teaching (TACT).

The final TACT canvas adopts a linear structure comprising 6 dimensions, broken down into a total of 16 guiding questions that deconstruct the overarching concept of trust into smaller, manageable components (Figure 1). French, German, Italian, and Spanish versions of TACT are provided in Multimedia Appendices 6-9, with the downloadable high-quality English version of TACT provided in Multimedia Appendix 10.

The first dimension, *Context*, reflects the context-specific nature of trust and prompts reflection on the historical and present circumstances shaping the trust relationship. This involves examining the trustor’s past experiences, current perceptions, and expectations of the trustee and the temporal frame in which the trust relationship unfolds. The relational nature of trust is addressed in the second dimension, *Actors*, which maps the key stakeholders involved in the trust dynamic—namely, the trustor, the trustee, and any additional actors who may influence trust. This dimension also prompts reflection on potentially vulnerable parties within the trust relationship, highlighting the need for tailored trust-building strategies. The third and fourth dimensions, *Causes* and *Effects*, are informed by the causal and effect dimensions identified in the “public trust in the health care system framework*”* [29]. These dimensions prompt analysis of the principles that influence the development of trust, as well as the potential positive and negative effects resulting from it. The fifth dimension, *Implications*, highlights that trust does not arise spontaneously; it requires continuous and deliberate efforts to be built and maintained over time. Once the causes and effects of trust are identified, it is necessary to determine the actions needed to promote these trust-building principles, as well as those that could potentially undermine trust, in order to actively safeguard it. This includes allocating the necessary resources and identifying appropriate evaluation methods to assess the effectiveness of the trust-building measures. Finally, *Reflections* prompts students to reflect critically on whether trust is indeed the most appropriate concept to apply in the given case study. This includes evaluating the potential consequences of a lack of trust and, if relevant, considering alternative concepts—such as confidence, reliance, or faith—that may offer a more suitable analytical lens.

### Tutorial on TACT

Below is an example of the application of TACT, presented as a written trust analysis of the case study used with MSc students and supported by references to relevant literature. This serves three main purposes as follows: (1) to show the practical application of TACT, (2) to provide readers with an explanation of each dimension and the guiding questions, and (3) to offer lecturers academic resources for teaching trust. The trust analysis below is not intended to be exhaustive or definitive; the case discussion reflects selected classroom exchanges and illustrative reasoning rather than a comprehensive assessment. Its purpose is to demonstrate how the canvas can be applied in practice.

#### Conducting a Trust Analysis of a Case Study by Using TACT

The case study was sourced from the Digital Society Initiative Strategy Lab 2022, *Level “Now.” Artificial Intelligence in Medicine—Case Study: “Diagnosis”* [37], and it was slightly adapted by FZ and FG for conciseness.

##### Background

Depression is a major health challenge, impacting up to 20% of the population and potentially leading to suicide (10%‐15%). Diagnosis can be difficult due to similarities with other psychological conditions such as low mood or sadness. Innovative behavioral observation methods aim to identify severe depression or high-risk individuals to prevent suicide. Social network interactions increasingly shape our daily lives, revealing insights into mental health through information consumption, likes, shares, and connections.

DeDe is an app developed by an interdisciplinary consortium of researchers and approved by the cantonal data protection authority, capable of reliably identifying individuals at risk of severe depression based on their social media behavior. Social media providers can integrate DeDe as an option for users, with evaluations kept confidential from the providers. Health insurance may compensate providers for offering DeDe, as early depression diagnosis reduces health care costs, with DeDe financed by insurance premiums.

##### Case

Alice, prone to depression, discovers DeDe through her primary care physician and downloads the app. During “low” periods, she turns to news about the uncertain world, exacerbating her mood. DeDe notices this and suggests lighter news options, sometimes effective. However, as Alice isolates herself and her mood worsens, DeDe alerts her to seek help from her brother, Peter, listed in the app.

Despite efforts, Alice often ignores recommendations and sinks deeper into depression, as evidenced by her lack of physical activity tracked by DeDe. When her psychiatrist, Sabine, receives an alert, she intervenes, convincing Alice to seek immediate outpatient psychiatric care.

Through therapy and medication, Alice’s depression is managed, averting a possible suicide.

### 1. Context

Trust is context-specific, shaped by past experiences, present perceptions, and future anticipations toward a benefit [5].

#### What Is the Present Context Trust Is Unfolding In?

Public trust develops in the public sphere through open public discourse on current perceptions of a system’s trustworthiness [5]. It is therefore essential to analyze the present context—whether social, economic, and political—in which trust is unfolding. This can be achieved by assessing news narratives, expert opinions, policies, and public perceptions of what is currently being evaluated [38].

#### Case Study

The present context (Switzerland, 2024) is characterized by the ongoing digital transformation of healthcare, alongside growing societal awareness of the importance of mental health.

#### What Is the Historical Context Influencing Present Trust Building?

Familiarity and shared past experiences are considered significant determinants of present public trust [5]. The close relationship between past experiences and trust is described by a trust culture arising from “the collective and shared experiences of societal members over time,” defining trust culture as “a product of history” [1]. Conferring trust requires consideration of all previous experiences, as trust can only be placed in a familiar world with a reliable background [30]. By collecting news articles, expert opinions, and policy documents from the past, it is possible to trace the evolution of the discourse and identify events that may have positively or negatively influenced current public trust levels [38].

#### Case Study

Past national scandals involving digital health apps (eg, the MyVaccination scandal in 2021) have shaped public narratives and perceptions on health information sharing, increasing skepticism. There are concerns about potential insurance adjustments based on a mental health diagnosis.

#### What Is the Timeframe the Trust-Relationship Is Unfolding In? Short-Term (Wks), Mid-Term (Mo), and Long-Term (Y)

Time plays a crucial role in trusting relationships, as trust cannot be rushed [5]. Establishing the timeframe for a trust relationship is essential for effective planning. The duration influences the dynamics of trust development, the factors that determine trustworthiness, and the resilience of the trust relationship over time. Short-term trust often relies heavily on immediate actions and clear communication, whereas long-term trust requires the development of deeper relational bonds. Analyzing the timeframe provides a more nuanced understanding of the strategies required to maintain or restore it.

#### Case Study

Trust begins when Alice opens up to her doctor and shares her struggles. Following the establishment of trust with her psychiatrist, Alice follows her recommendation, downloads the app, uses its features, and responds to alerts prompting timely interventions (short term, weeks). Sustained trust in the long term (years) among Alice, Sabine, and Peter is crucial, as DeDe supports Alice’s ongoing mental health management while maintaining trust in its privacy safeguards, effectiveness, and integration into healthcare systems.

### 2. Actors

#### Who Trusts Whom?

Trust is a relational concept where A trusts B to do or not do something. The relational nature of trust means it is influenced by the actions and behaviors of the involved actors [5]. Mapping “who trusts whom” is of fundamental importance to ensure a clear representation of the trust network and to highlight existing dependencies.

#### Case Study

Alice trusts her psychiatrist, Sabine, to recommend the DeDe app as an appropriate tool for managing her care. She also trusts her brother Peter to provide support and intervene if she is in distress when alerted by the app. Sabine trusts that the DeDe app was developed in a way that accurately monitors Alice’s mental health, detects any deterioration, and notifies her when intervention is necessary. Sabine also trusts Alice to use the app responsibly and Peter to respond appropriately when needed.

#### Who Is Vulnerable in the Trust Relationship and to What Extent?

Trust arises in contexts characterized by complexity, risk, and vulnerability [5]. In certain circumstances, specific groups may be more vulnerable than others within trust relationships. For instance, individuals with low digital literacy might struggle to engage effectively with the digital health intervention [39].

It is therefore essential to identify who is vulnerable in this context as this helps determine whether trust is the appropriate analytical concept to apply.

#### Case Study

Alice is particularly vulnerable as, trusting her doctor’s advice, she has downloaded the DeDe app to receive effective and timely recommendations. However, this requires her to share highly sensitive personal information, exposing her to potential risks related to data privacy and misuse.

#### Who Can Influence the Trust Relationship?

Trust is a relational construct that requires at least one other party in whom trust is placed, though it often involves multiple actors [5]. It is crucial not only to map “who trusts whom” but also to identify who can influence the trust relationship. These influencing factors may include individuals, institutions, or broader dynamics that shape the trust relationship.

#### Case Study

Cantonal data protection authority: By approving DeDe, the cantonal data protection authority reassures users about data security and privacy, fostering institutional trust. As a health care professional, Sabine influences trust through her endorsement and use of DeDe. Peter’s role as a trusted contact can influence Alice’s use of the app. If he responds positively to alerts, this reinforces Alice’s trust in DeDe as a supportive tool.

### 3. Causes: What Builds Trust?

Familiarity, the presence of an active regulatory system, anonymization of personal data, offering autonomous choices, truthful and honest communication about health system activities, protecting privacy, demonstrating the capacity to fulfill the entrusted action, ensuring data security, certainty about the future, anticipation of a net benefit as an outcome of the trust relationship, and sufficient time for those handling the entrusted data are among the trust-building principles identified in “public trust in the health system” [29] and “public trust in health data sharing” frameworks [28]. These principles are broadly valid within the realms of health data sharing and health system initiatives. However, the specific principles that “build trust” in a given case study must be carefully selected and justified based on the context.

#### Case Study

Through communication, Sabine provides detailed information on the app’s functionalities, as well as the associated risks and benefits of using it, enabling Alice to make an informed decision. Robust data security ensures that Alice feels safe sharing sensitive personal information, addressing concerns about privacy and fostering confidence in the app. The perceived personal benefit of the app to Alice’s well-being, such as mood improvement and mental health support, incentivizes her to download DeDe and use the app. All of the above may be necessary to overcome skepticism toward digital health solutions involving the sharing of health data, particularly in light of the MyVaccination scandal.

### 4. Effects

#### What Are the Potential Positive Effects of the Trust Relationship on Involved Actors?

Health system legitimization and public acceptance are the results of a strong public-system trust relationship. Moreover, trust reduces uncertainty, streamlines decision-making processes, and promotes long-term partnerships. Additionally, trust may lead to greater efficiency, as it lowers the need for constant oversight and can result in mutual benefits such as shared learning, innovation, and the achievement of common goals [5].

#### Case Study

The use of the DeDe app facilitates timely intervention, leading to improved mental health, greater stability, and better management of Alice’s depression. Trust fosters stronger relationships between Alice, her psychiatrist Sabine, and Peter, enabling coordinated support for her well-being.

#### What Are the Potential Negative Effects of the Trust Relationship on Involved Actors?

While trust can be beneficial, it may also carry potential risks and negative effects. Over-reliance on trust can make actors vulnerable to breaches or exploitation, particularly when trust is misplaced or taken for granted. This may lead to significant consequences such as reduced vigilance or a lack of accountability. Furthermore, if trust is broken, the resulting loss can damage relationships [5,40].

#### Case Study

Overreliance on the DeDe app may lead to a decline in direct personal interactions between Alice, her psychiatrist Sabine, and her brother Peter, potentially weakening the human aspect of care and support. If the app fails to identify severe episodes or provide inaccurate recommendations, Alice’s condition could worsen, undermining her confidence in digital tools and health care systems.

### 5. Implications

Trust does not emerge by itself; it requires continuous and targeted efforts to be built and sustained over time. Once the principles that contribute to “building trust” have been identified, it is essential to determine the actions needed to promote these trust-building principles, as well as those that could potentially undermine trust, to actively safeguard it. To achieve this, it is also necessary to allocate the required resources and identify appropriate evaluation methods to measure whether the trust-building actions have been successful.

#### On the Basis of the Causes, What Actions Promote Trust?

For instance, if we consider the principle of an “active regulatory system”, the presence of legislation, policies, and laws can be seen as trust-promoting activities. Similarly, in the case of the “communication” principle, establishing a targeted communication campaign serves as a viable trust-building activity [28].

#### Case Study

Providing detailed information about the app’s functionalities, as well as its risks and benefits, enables Alice to make an informed decision and fosters trust in the health care provider and confidence in the app. Implementing robust data security measures addresses concerns about privacy, ensuring Alice feels safe and confident sharing sensitive personal information.

#### On the Basis of the Causes, What Actions Endanger Trust?

On the contrary, a lack of effective communication can undermine trust, as it prevents stakeholders from accessing essential information about an initiative. Information is the lifeblood of trust; therefore, the absence of clear communication and information-sharing jeopardizes trust-building efforts [5].

#### Case Study

If Sabine fails to provide detailed information about the app’s functionalities, as well as its risks and benefits, Alice may feel uncertain and could decide not to download the app. Insufficient protection of sensitive personal information can raise concerns about privacy breaches, undermining Alice’s confidence in the app. If the app fails to deliver the expected benefits, her confidence in the app and trust in the health care professional may diminish.

#### Which Resources Are Needed to Build Trust?

Based on the causes of trust, a variety of resources are required for implementation. These include time, financial investment, and human resources, among others.

#### Case Study

Sufficient funding is needed to ensure regular app updates, data security measures, ethical evaluations, and to develop educational materials, outreach programs, and communication strategies.

#### How to Evaluate Trust-Building Actions?

Research indicates that the evaluation and measurement of trust remain challenging tasks, requiring methodological creativity [41]. Nevertheless, evaluation, including robust measurement, is critical to understanding a health system’s capacity to build and sustain trust. A common method for collecting trust-related data is the use of survey instruments [42] complemented by qualitative indicators (eg, user testimonials) or by monitoring the expected effects of trust, such as participation or engagement with a digital solution.

#### Case Study

Indicators of user engagement, such as the frequency with which users interact with the app, respond to alerts, or act on its recommendations, may serve as proxies for trust.

### 6. Reflections

On the basis of the analysis conducted in points 1 to 5, students are asked to reflect on whether trust is ultimately the appropriate concept to apply to the given case study. They should evaluate the consequences of a lack of trust and, if necessary, propose alternative concepts that may be more suitable (eg, confidence, reliance, or faith).

#### Why Is Trust Worthy of Consideration?

##### Case Study

Trust fosters collaboration between users (Alice), family members (Peter), and professionals (Sabine), ensuring that the support network functions effectively, while confidence in data security and privacy facilitates Alice’s use of the app.

### What Would Be the Case Without Trust?

#### Case Study

Without trust, Alice might not have followed her doctor’s recommendation, downloaded the app, or shared her data, thereby reducing the feasibility and effectiveness of early detection and intervention and increasing the risk of crisis escalation or suicide.

### Are There More Suitable Concepts Other Than Trust?

#### Case Study

Trust is a relevant concept in the relationship between Alice, Sabine, and Peter. When it comes to downloading and using the app, the concept of confidence may be more accurate, as Alice needs to be confident that DeDe will perform as expected, keep her data secure, and maintain privacy.

## Discussion

To our knowledge, TACT is the first canvas specifically designed to support students across disciplines and academic levels in analyzing trust-related issues in digital public health and medicine, by bridging theory and practice, and facilitating the application of conceptual knowledge to real-world scenarios. Feedback collected from students indicates that TACT met its core objective: to provide a structured and accessible framework that enables students to understand complex concepts of trust, simplify trust analysis, and apply these insights to practical case studies—thereby enhancing both teaching practices and student learning outcomes. Its adaptability to different pedagogical approaches (guided and problem-based learning) and learning environments (in-person and online), as well as its suitability for use in both digital and printed formats, further reinforces its value as a teaching tool. TACT offers educators a structured framework for teaching trust and provides students with a practical, engaging tool for conducting trust analyses of digital public health and medicine case studies.

During testing, students reported that TACT effectively fostered an understanding of trust across both teaching approaches by logically guiding them in considering different aspects of trust. When applied within the guided learning approach, TACT offered a well-informed and theoretically grounded framework, enabling students to engage with the canvas based on prior conceptual input. However, this approach may risk fostering a more passive learning experience, as students tend to depend on predelivered content rather than actively constructing their own understanding. Conversely, the problem-based learning approach encourages critical thinking and problem-solving skills, potentially leading to deeper engagement through self-directed exploration. However, its effectiveness relies on a carefully designed and time-intensive debriefing process to clarify conceptual ambiguities and consolidate learning. These observations align with Kirschner et al’s [33] argument that guided instruction becomes less necessary when learners possess sufficient prior knowledge to engage with material independently. Consequently, problem-based learning may be more suitable for advanced students, such as MSc and PhD candidates, who possess stronger critical and independent thinking skills. In contrast, undergraduate students may benefit more from guided learning, which offers additional structure and foundational knowledge to support their learning process.  

While the use of canvases has been shown to facilitate interactive and participatory learning, fostering active problem-solving, critical thinking, deeper understanding, and long-term retention of course material [23], literature highlights the need to address potential challenges related to content accessibility and readability. Students with dyslexia, for instance, may struggle with complex text structures, underscoring the importance of clear, structured, and visually accessible materials [43]. Similarly, non-native English speakers may encounter difficulties with academic texts that use advanced vocabulary or idiomatic expressions, potentially hindering their learning process [44]. Additionally, variations in academic preparedness across students further complicate this issue, as learners from diverse educational backgrounds may have different levels of experience with critical reading and text analysis, necessitating materials that accommodate a broad spectrum of reading abilities [23]. To enhance clarity, accessibility, and usability, TACT was structured as linearly as possible, minimizing lengthy text and prioritizing plain English. It was tested with both native and non-native English speakers across academic levels, from undergraduate to PhD students, ensuring that its content and structure were clear, logical, and intuitive. Additionally, the color palette was selected to be suitable for color-blind users, and icons were incorporated as complementary visual aids rather than essential informative elements to reduce the risk of misinterpretation. While these refinements improve accessibility, lecturers may still need to make ad hoc adjustments to accommodate individual student needs.

The use of case studies in education aligns with constructivist theory and follows the tradition of active and experiential learning, supporting students in developing critical thinking and decision-making skills alongside domain-specific knowledge [45-47]. While TACT is grounded in the authors’ conceptual research on trust in digital health and policy and has thus far been tested only on digital public-health case studies, the deliberate effort to ensure its dimensions are discipline-independent, combined with the canvas’s flexibility in case selection, supports its broader relevance and adaptability. As TACT could guide the analysis of trust in case studies from diverse domains, it offers lecturers the flexibility to tailor its use by selecting cases aligned with their students’ learning objectives. Students may likewise apply TACT to their own research projects, thereby enhancing engagement and learning impact [48]. We encourage future research to test the applicability of TACT beyond case studies on digital public health and medicine, and in other geographical regions to further demonstrate its transferability and adaptability. Moreover, assessing the durability of students’ conceptual understanding of trust and their ability to apply it across different contexts over time represents a valid direction for future research.

The primary goal of TACT is not to provide definitive answers but to stimulate critical thinking and discussion on trust. To achieve this, we deliberately avoided checklists or predefined answers, encouraging students to explore multiple perspectives and identify the most appropriate answers for their specific case studies. Lecturers can use the literature provided to deepen their understanding of trust and its underlying principles. The flexibility of TACT extends across disciplines, academic levels, and teaching formats. It can be used for both high-level and in-depth trust analyses, ranging from a quick exploratory tool that prompts consideration of various trust-related aspects in a case study to a systematic framework for comprehensive trust analysis, where each of the 6 dimensions can be explored in dedicated lectures, allowing for an in-depth study of the theoretical foundations. The decision on how to integrate TACT into teaching remains at the discretion of lecturers, who can adapt its use to align with students’ curricula and academic needs.

## Supplementary material

10.2196/79709 Multimedia Appendix 1 Initial version of the canvas reviewed by senior researchers.

10.2196/79709 Multimedia Appendix 2 Adapted AI in medicine “Diagnosis” case study used in MSc focus group (Digital Society Initiative Strategy Lab 2022).

10.2196/79709 Multimedia Appendix 3 COVID-19–based case study developed by authors and used in BSc online focus group.

10.2196/79709 Multimedia Appendix 4 Case study developed by authors based on recent Swiss health policy developments and used in PhD online focus group.

10.2196/79709 Multimedia Appendix 5 Comparison of initial and final canvas versions highlighting five key changes.

10.2196/79709 Multimedia Appendix 6 Trust analysis canvas–teaching (French).

10.2196/79709 Multimedia Appendix 7 Trust analysis canvas–teaching (German).

10.2196/79709 Multimedia Appendix 8 Trust analysis canvas–teaching (Italian).

10.2196/79709 Multimedia Appendix 9 Trust analysis canvas–teaching (Spanish).

10.2196/79709 Multimedia Appendix 10 Trust analysis canvas–teaching (TACT) (English).

10.2196/79709 Checklist 1 COREQ checklist.

## Abbreviations

AI: artificial intelligence
COREQ: Consolidated Criteria for Reporting Qualitative Research
TACT: Trust Analysis Canvas for Teaching
UZH: University of Zurich
