# Supplementary figures and images for: Trust Analysis Canvas for Teaching in the Field of Digital Public Health and Medicine: Tutorial
Source: JMIR Med Educ. 2026 Feb 17;12:e79709. doi: 10.2196/79709 (PMC12912458; doi:10.2196/79709)

# Appendix 1

Initial version of the canvas reviewed by senior researchers:


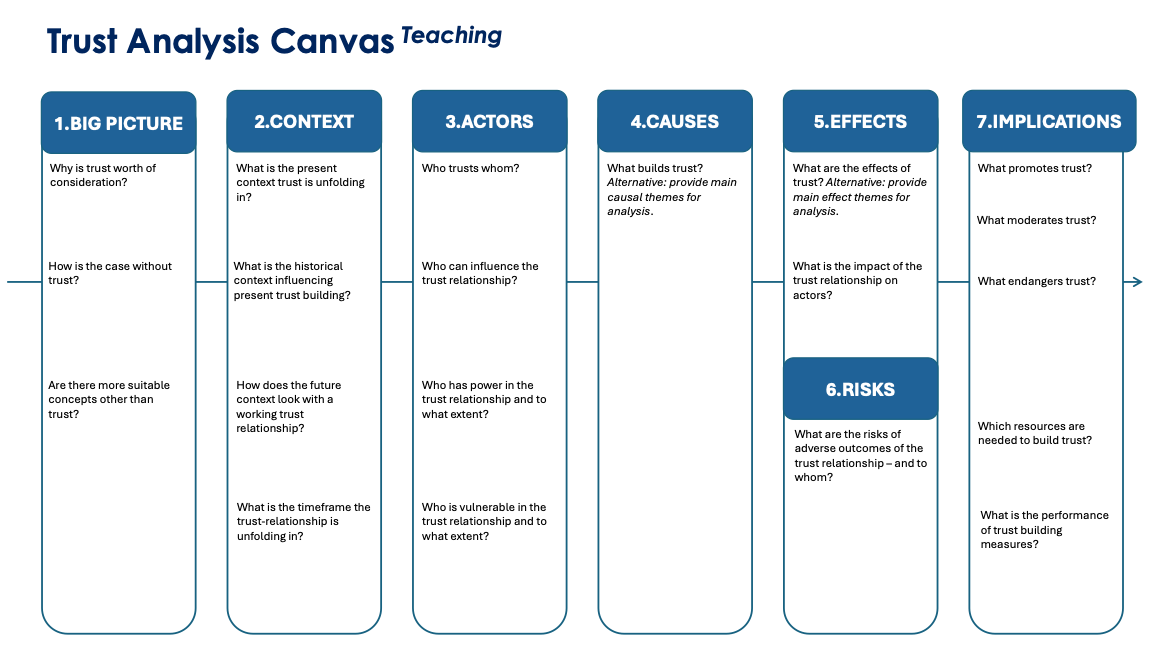

Supplement: Multimedia Appendix 1 [file mededu-v12-e79709-s001.docx]

# Multimedia Appendix 6

#
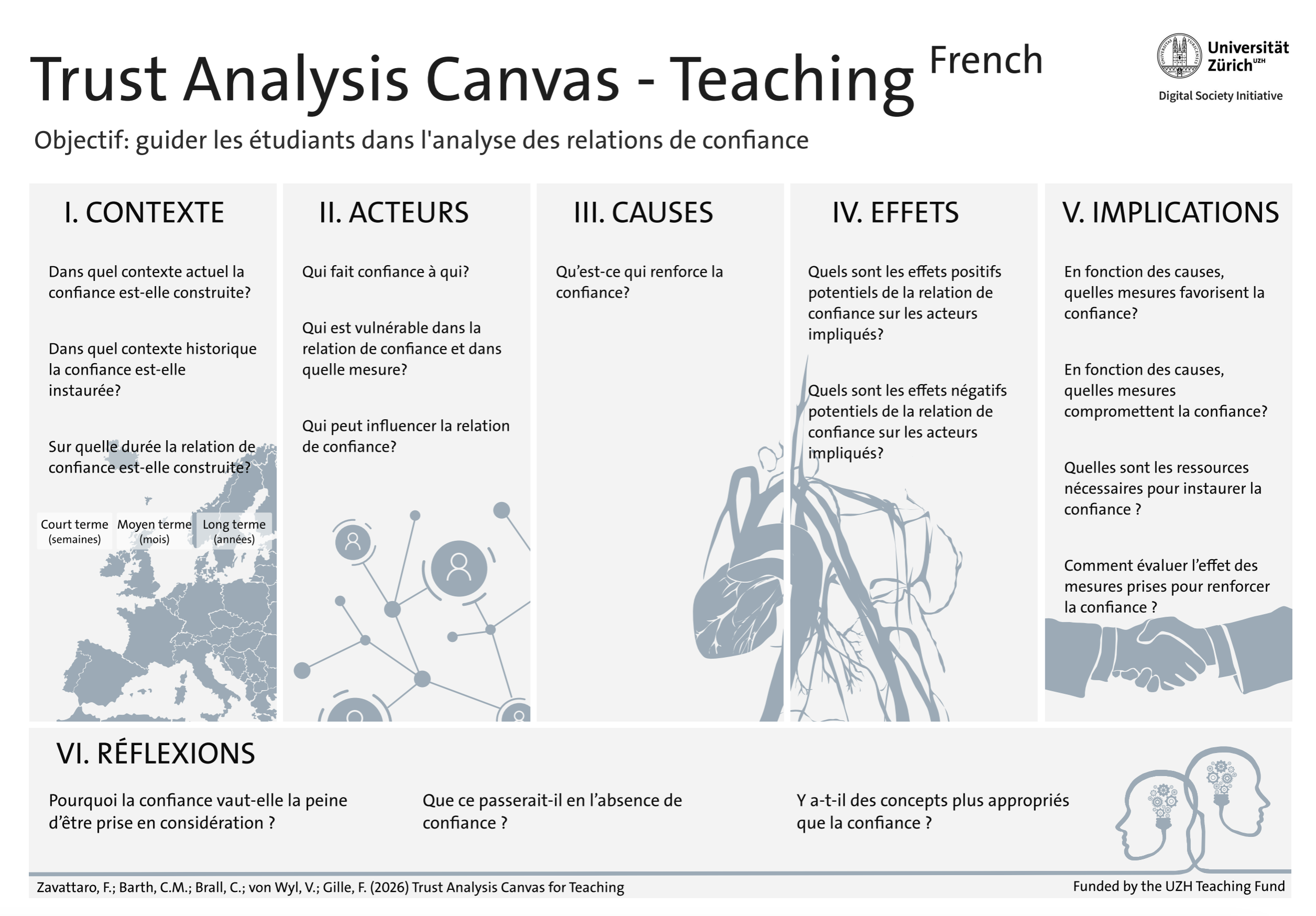

Supplement: Multimedia Appendix 6 [file mededu-v12-e79709-s006.docx]

# Multimedia Appendix 7
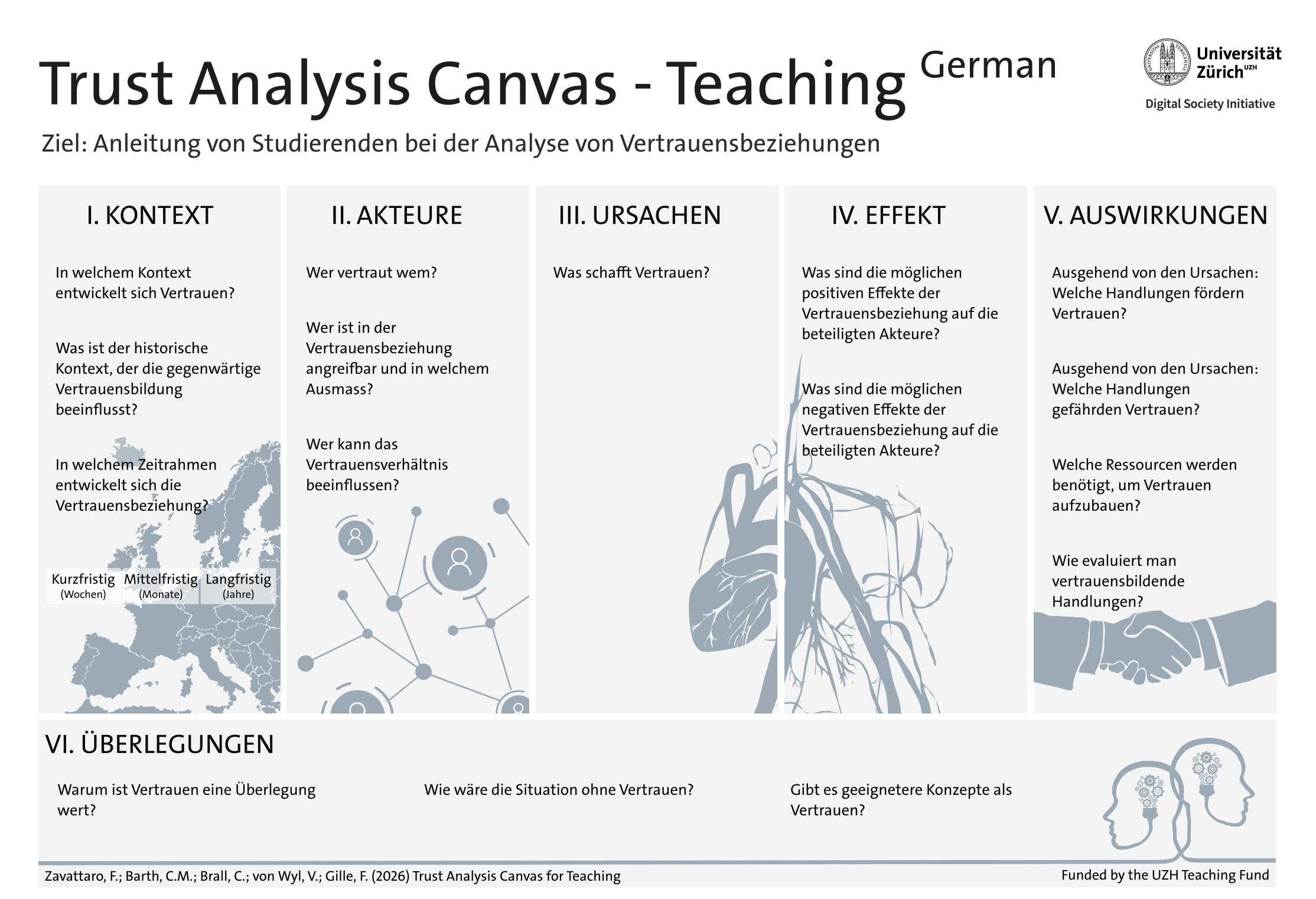

Supplement: Multimedia Appendix 7 [file mededu-v12-e79709-s007.docx]

# Multimedia Appendix 8

#
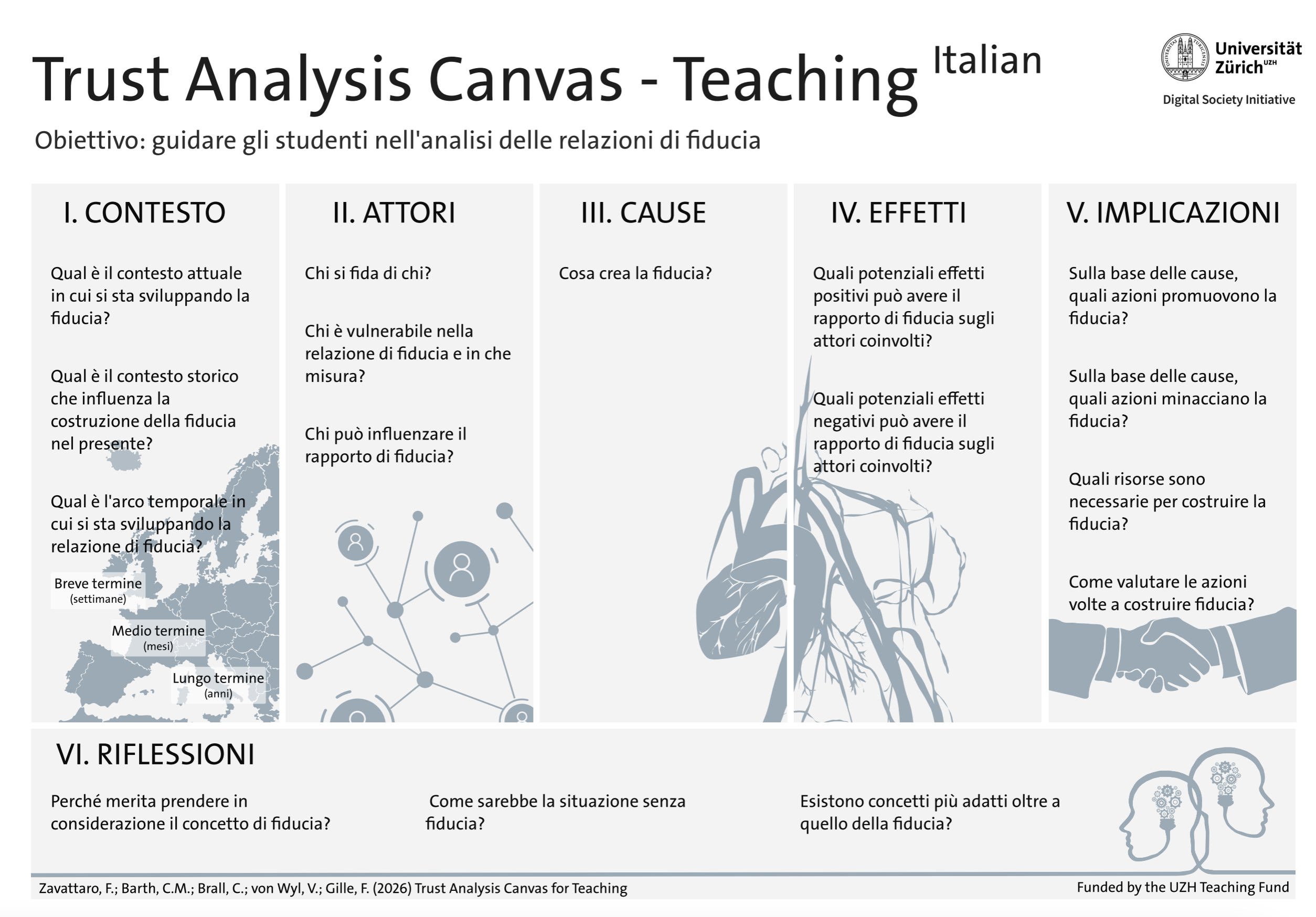

Supplement: Multimedia Appendix 8 [file mededu-v12-e79709-s008.docx]

# Multimedia Appendix 9


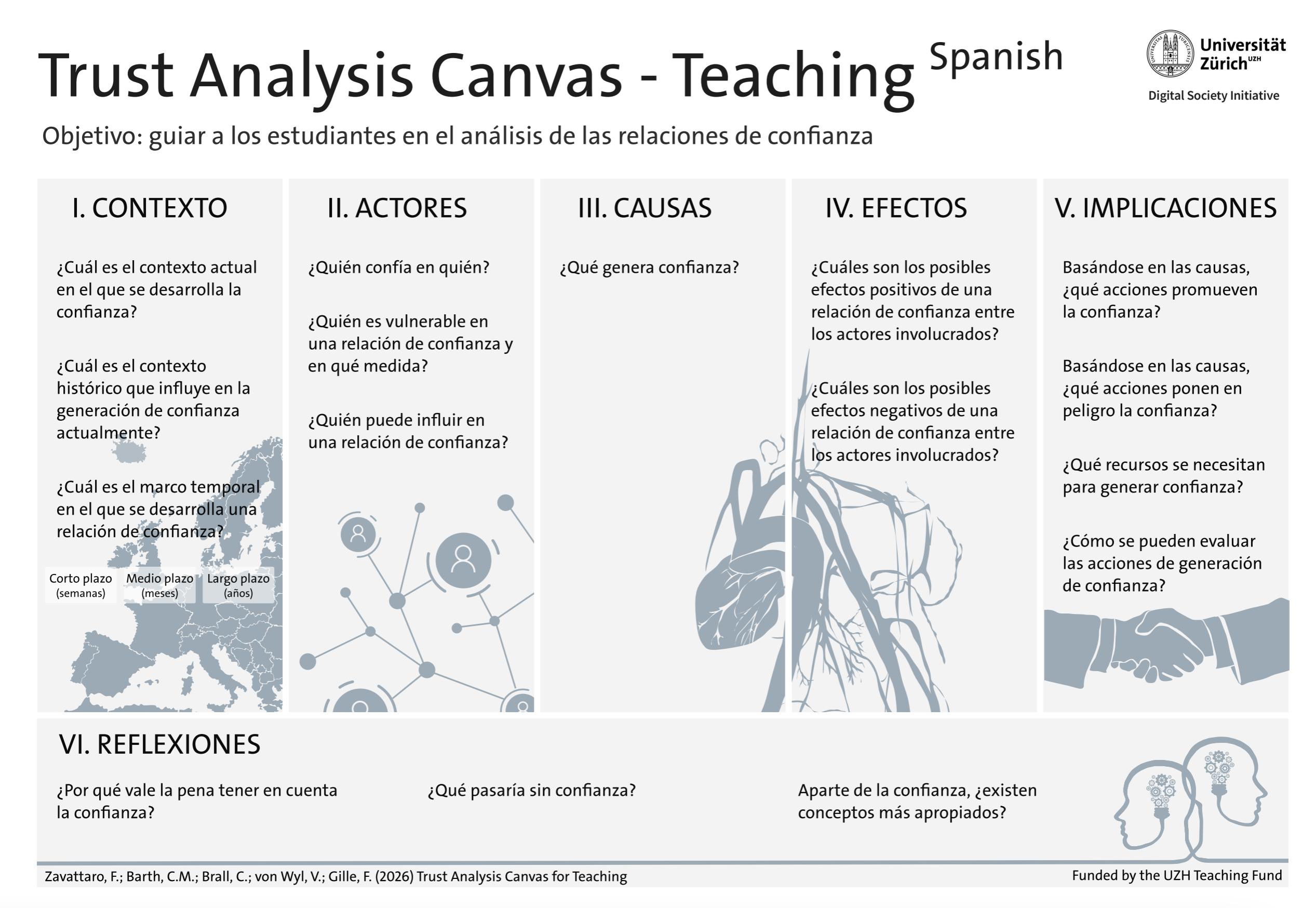

Supplement: Multimedia Appendix 9 [file mededu-v12-e79709-s009.docx]
