# Supplementary material for: Trust Analysis Canvas for Teaching in the Field of Digital Public Health and Medicine: Tutorial
Source: JMIR Med Educ. 2026 Feb 17;12:e79709. doi: 10.2196/79709 (PMC12912458; doi:10.2196/79709)
Supplement: Multimedia Appendix 2 [file mededu-v12-e79709-s002.docx]

# Multimedia Appendix 2

The case study below was sourced from the Digital Society Initiative Strategy Lab 2022, Level ‘Now’. Artificial Intelligence in Medicine – Case Study: «Diagnosis» (C)[38]. It was slightly adapted by FZ and FG for conciseness and used in the in-person focus group conducted with MSc students.

**Background**

Depression is a major health challenge, impacting up to 20% of the population and potentially leading to suicide (10-15%). Diagnosis can be difficult due to similarities with other psychological conditions such as low mood or sadness. Innovative behavioral observation methods aim to identify severe depression or high-risk individuals to prevent suicide. Social network interactions increasingly shape our daily life, revealing insights into mental health through information consumption, likes, shares, and connections.

DeDe is an app developed by an interdisciplinary consortium of researchers and approved by the cantonal data protection authority, capable of reliably identifying individuals at risk of severe depression based on their social media behavior. Social media providers can integrate DeDe as an option for users, with evaluations kept confidential from the providers. Health insurances may compensate providers for offering DeDe, as early depression diagnosis reduces healthcare costs, with DeDe financed by insurance premiums.

**Case:**

Alice, prone to depression, discovers DeDe through her primary care physician and downloads the app.

During ‘low’ periods, she turns to news about the uncertain world, exacerbating her mood. DeDe notices this and suggests lighter news options, sometimes effective. However, as Alice isolates herself and her mood worsens, DeDe alerts her to seek help from her brother, Peter, listed in the app.

Despite efforts, Alice often ignores recommendations and sinks deeper into depression, evident by her lack of physical activity tracked by DeDe. When her psychiatrist, Sabine, receives an alert, she intervenes, convincing Alice to seek immediate outpatient psychiatric care.

Through therapy and medication, Alice's depression is managed, averting a possible suicide.
