# Supplementary material for: Trust Analysis Canvas for Teaching in the Field of Digital Public Health and Medicine: Tutorial
Source: JMIR Med Educ. 2026 Feb 17;12:e79709. doi: 10.2196/79709 (PMC12912458; doi:10.2196/79709)
Supplement: Multimedia Appendix 3 [file mededu-v12-e79709-s003.docx]

# Multimedia Appendix 3

The case study below was developed by FZ and FG based on recent experience with the Covid-19 pandemic[50] and was employed in the online focus group held with BSc students.

“On 19 December 2020, the Swiss Agency for Therapeutic Products (Swissmedic) approved the Pfizer-BioNTech COVID-19 vaccine for regular use. Following this approval, all citizens residing in Switzerland were progressively given the opportunity to receive the vaccination if they chose to do so.

The COVID-19 pandemic period was marked by contrasting opinions on how to combat the virus. The public had to decide whether to trust the advice of doctors who recommended getting vaccinated.

**Task**: run a trust analysis to identify key factors that influenced doctor-patient trust during the COVID-19 vaccination campaign.”
