# Supplementary material for: Trust Analysis Canvas for Teaching in the Field of Digital Public Health and Medicine: Tutorial
Source: JMIR Med Educ. 2026 Feb 17;12:e79709. doi: 10.2196/79709 (PMC12912458; doi:10.2196/79709)
Supplement: Multimedia Appendix 4 [file mededu-v12-e79709-s004.docx]

# Multimedia Appendix 4

The following case study was developed by FZ and FG, based on recent developments in the Swiss health policy landscape[51], and was employed in the online focus group held with PhD students.

“The Swiss government, with the ‘DigiSanté’ program 2025-2034, aims to address Switzerland’s delay in digitizing its healthcare system. A key component of the program is the implementation of the Electronic Patient Dossier (EPD) to securely collect, store, and exchange health information between patients and their healthcare professionals.

To successfully implement this initiative public trust is needed as, without trust, citizens won’t open the EPD.

**Task**: Perform a trust analysis using the TACT to identify key variables influencing public trust in this discourse.
