# Supplementary material for: Trust Analysis Canvas for Teaching in the Field of Digital Public Health and Medicine: Tutorial
Source: JMIR Med Educ. 2026 Feb 17;12:e79709. doi: 10.2196/79709 (PMC12912458; doi:10.2196/79709)
Supplement: Multimedia Appendix 5 [file mededu-v12-e79709-s005.docx]

# Multimedia Appendix 5

A comparison between the initial and final versions of the canvas reveals five key changes. First, the number of main dimensions was reduced from seven to six by integrating the dimension ‘*Risk’* into ‘*Effect’* to avoid redundancy. Second, ‘*Big Picture’* was rephrased as ‘*Reflections’* and repositioned as the final dimension, enabling students to evaluate the relevance of trust at the end of the analysis. Third, the layout shifted from a linear left-to-right format to a more structured design, with ‘*Reflections’* spanning horizontally below the five dimensions to emphasize its role as a concluding reflection step. Fourth, the phrasing of some of the guiding questions was refined for clarity and precision, reducing potential misunderstandings. Finally, the overall visual design was improved, resulting in a more professional appearance. Icons of the final canvas have been carefully selected to establish a visual connection to each dimension while reflecting the tool’s interdisciplinary nature. ‘*Context’* is represented by a map, symbolizing the geopolitical domain, while ‘*Actors’* is depicted by a network icon, linking to the field of communication science. ‘*Causes’* and ‘*Effects’* are illustrated with a heart and vascular system, representing the health and scientific disciplines, whereas ‘*Implications’* are conveyed through a handshake, reflecting the social sciences. ‘*Reflections’* is symbolized by two thinking brains, referring to the philosophical domain.
