# Supplementary material for: Trust Analysis Canvas for Teaching in the Field of Digital Public Health and Medicine: Tutorial
Source: JMIR Med Educ. 2026 Feb 17;12:e79709. doi: 10.2196/79709 (PMC12912458; doi:10.2196/79709)
Supplement: Multimedia Appendix 10 [file mededu-v12-e79709-s010.pdf]

Trust Analysis Canvas - Teaching

Universität Zürich[UZH]
Digital Society Initiative

Aim: to guide students in analysing trust relationships

I. CONTEXT

What is the present context trust is unfolding in?

What is the historical context influencing present trust building?

What is the timeframe the trust-relationship is unfolding in?

Short term (weeks)	Mid term (months)	Long term (years)

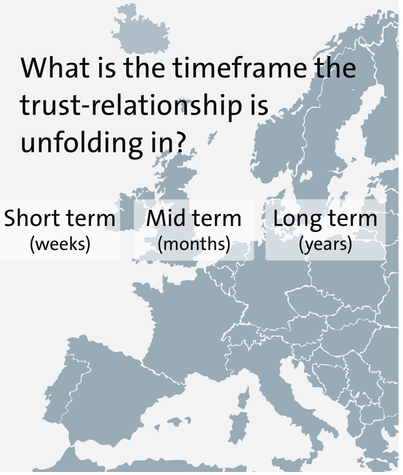

II. ACTORS

Who trusts whom?

Who is vulnerable in the trust relationship and to what extent?

Who can influence the trust relationship?

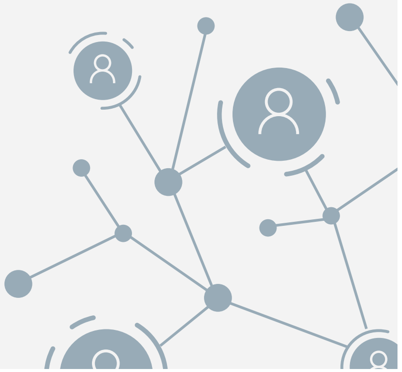

III. CAUSES

What builds trust?

IV. EFFECTS

What are the potential positive effects of the trust relationship on involved actors?

What are the potential negative effects of the trust relationship on involved actors?

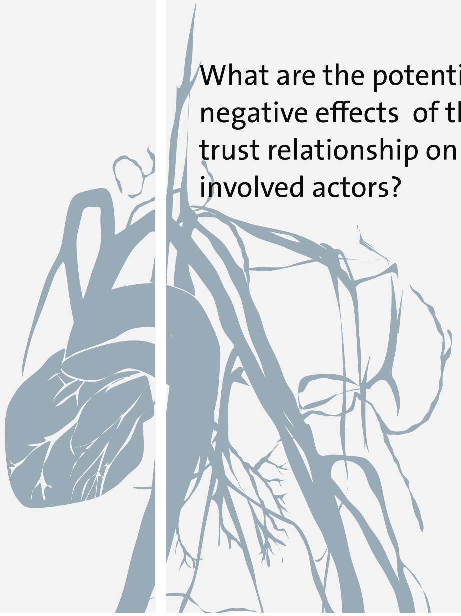

V. IMPLICATIONS

On the basis of the causes, what actions promote trust?

On the basis of the causes, what actions endanger trust?

Which resources are needed to build trust?

How to evaluate trust building actions?

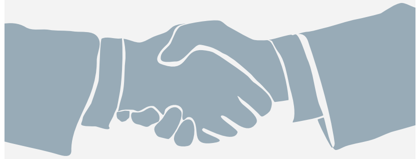

VI. REFLECTIONS

Why is trust worth of consideration?

What would be the case without trust?

Are there more suitable concepts other than trust?

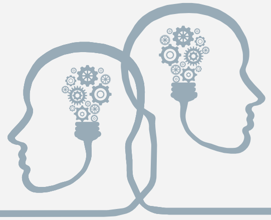

Zavattaro, F.; Barth, C.M.; Brall, C.; von Wyl, V.; Gille, F. (2026) Trust Analysis Canvas for Teaching

Funded by the UZH Teaching Fund
